# The Dangerous Drugs Act 1920 and Hospitals

**Published:** 1920-10-16

**Authors:** 


### The Dangerous Drugs Act 1920 and Hospitals.

THE position of pharmacists engaged at public institutions under the above-mentioned Act is at present very ill-defined, for whilst the statement is made that regulations *shall* provide for "authorising" any person who lawfully keeps an open chemist's shop, no mention is made that qualified pharmacists, as such, will be authorised persons to keep and distribute, under medical prescriptions, the drugs mentioned in the Act. Unless a definite assurance is given that institutional pharmacists are to have equal rights with their colleagues in retail business, it would seem necessary for every institutional pharmacist to take out a licence.

It will be remembered that when the Cocaine and Opium Regulations were made under the Defence of the Realm Act the same anomaly existed, for, under that Act, pharmacists carrying on *retail* business were "authorised" persons, whilst their hospital and institutional colleagues, with an equal qualification, were acting illegally in dispensing preparations of cocaine. The absurdity of the position was made evident to the Home Office, who made the regulations, and a general licence was promptly issued making persons "duly qualified under the Pharmacy Acts and who were engaged at public institutions" authorised persons under the Defence of the Realm Act.

We note by a report of a meeting of the Council of the Pharmaceutical Society of Great Britain that they have already received representations on the matter from the Public Pharmacists' Association, and the Council has decided to support the claim of institutional pharmacists to become authorised persons, without the necessity of taking out a licence.

